# Unraveling a Subgroup of Men With Unexplained Male Infertility—Men With Normogonadotropic Nonobstructive Azoospermia

**DOI:** 10.1210/clinem/dgaf200

**Published:** 2025-04-15

**Authors:** Vanessa Schwarzkopf, Joachim Wistuba, Reinhild Sandhowe-Klaverkamp, Sabine Kliesch, Jörg Gromoll, Maria Schubert

**Affiliations:** Centre of Reproductive Medicine and Andrology, Institute of Reproductive and Regenerative Biology, University of Münster, Münster 48149, Germany; Centre of Reproductive Medicine and Andrology, Institute of Reproductive and Regenerative Biology, University of Münster, Münster 48149, Germany; Centre of Reproductive Medicine and Andrology, Institute of Reproductive and Regenerative Biology, University of Münster, Münster 48149, Germany; Centre of Reproductive Medicine and Andrology, Department of Clinical and Surgical Andrology, University of Münster, EAA Training Center, Münster 48149, Germany; Centre of Reproductive Medicine and Andrology, Institute of Reproductive and Regenerative Biology, University of Münster, Münster 48149, Germany; Centre of Reproductive Medicine and Andrology, Department of Clinical and Surgical Andrology, University of Münster, EAA Training Center, Münster 48149, Germany

**Keywords:** nonobstructive azoospermia, follicle-stimulating hormone (FSH), spermatogenesis, unexplained infertility

## Abstract

**Context:**

Nonobstructive azoospermia (NOA) constitutes male infertility with complete absence of sperm in the ejaculate. NOA can originate in testicular malfunction or in endocrine dysregulation. Elevated follicle-stimulating hormone (FSH) levels are diagnostically valuable for NOA.

**Objective:**

An azoospermic patient cohort comprising 79 men and exhibiting no obstruction but normal FSH levels was identified. Focusing on this normogonadotropic nonobstructive azoospermic (NNOA) group, the study aimed to characterize these patients in depth.

**Methods:**

Whether the missing FSH upregulation in patients with NNOA is due to testicular or pituitary/hypothalamic malfunctions was examined by analyzing somatic, endocrine, and testicular parameters compared with 87 men with hypergonadotropic NOA and 88 normozoospermic men.

**Results:**

Testicular phenotypes of patients with NNOA and NOA were compared in histologically stratified subgroups (most advanced germ cell type). Using flow cytometry, the samples were evaluated for testicular cell composition by ploidy analysis. Concerning the distinct histological classification (hypospermatogenesis, spermatogenic arrest, Sertoli cell only, tubular atrophy) NNOA men produced more elongated spermatids and showed higher sperm retrieval. Testicular tissue composition between patients with NNOA and patients with NOA only differed after meiosis.

**Conclusion:**

The missing FSH upregulation in NNOA might be due to a testicular malfunction, as both FSH and testosterone were normal and NNOA spermatogenesis differed only after meiosis. Two explanations are possible: NNOA represents a phenotype in which spermatogenesis fails—different from NOA—only at the postmeiotic level, leaving FSH regulation unaffected, or the same mechanism underlies both NNOA and NOA, but the groups are at different stages of progression of the same disorder.

Infertility affects approximately 15% of couples in Western countries and in about 50% of the cases a male factor is an essential contribution (1). Various factors can provoke spermatogenic dysfunction (eg, oncological or genetic disorders); however, a strong etiologic factor can be identified only in around 28% of infertile men, while for the other 72% the causes remain unclear, which is termed unexplained if common semen parameters are affected or idiopathic in the case of normozoospermia (2).

The most severe phenotype of infertility is azoospermia, which is defined by the absence of sperm in the ejaculate. Azoospermia can be distinguished as obstructive azoospermia and nonobstructive azoospermia (NOA). In obstructive azoospermia, spermatogenesis might be intact but (eg, due to malformation or infections of the ductus deferens) sperm cannot be detected in the ejaculate, whereas in NOA, spermatogenesis is impaired (eg, caused by a failure of endocrine regulation or spermatogenic progress) and no sperm can be detected either (Fig. 1) (3). Azoospermic patients are routinely offered conventional testicular sperm extraction (TESE), or microdissection TESE (mTESE) followed by intracytoplasmic sperm injection when spermatozoa are obtained (4). However, detecting spermatozoa in the biopsies is a prerequisite for therapy and their presence is not always guaranteed as it depends on the histological composition of the tissues, which often reveals Sertoli cell only (SCO) syndrome, maturation arrest at different spermatogenic differentiation stages, or hypospermatogenesis (5). The different spermatogenic stages are dependent on the differentiation of germ cells from spermatogonia, via spermatocytes, round spermatids, to finally elongated spermatids, culminating in the formation of spermatozoa (6). Spermatogenesis can be disrupted for a variety of reasons at any level of this differentiation sequence, resulting in respective spermatogenic arrests (7). Histopathologically, this varies widely and the classification is based on the level at which spermatogenesis is arrested: spermatogonia (premeiotic), primary/secondary spermatocytes (meiotic arrest), or round spermatids (Fig. 1) (8).

**Figure 1.**
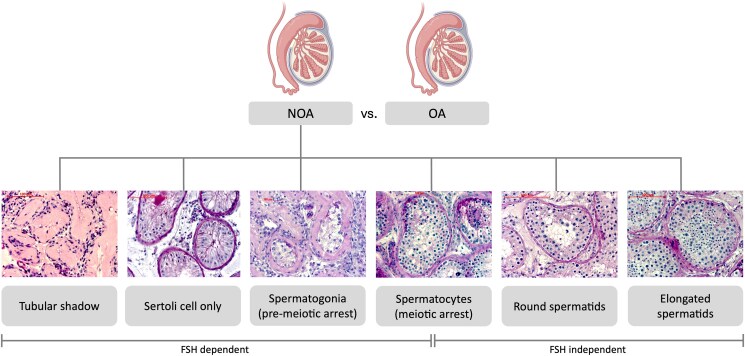
Histological phenotypes in NOA. Azoospermia can be divided into obstructive (OA) and nonobstructive azoospermia (NOA). Distinct histological patterns, reflecting the different developmental stages can generally be detected in NOA: tubular shadow, Sertoli cell only, spermatogonia (premeiotic), spermatocytes (meiotic), and round and elongated spermatids (postmeiotic). Spermatogenic arrest is classified based on the level at which spermatogenesis is arrested: spermatogonia (premeiotic), primary/secondary spermatocytes (meiotic arrest), or round spermatids. Early spermatogenesis is FSH dependent, while differentiation beyond the spermatocyte stage occurs independently of FSH. The representative histological images are scans of periodic acid–Schiff stainings that were conducted as part of the clinical routine at Centre of Reproductive Medicine and Andrology (CeRA) Münster.

A prerequisite for the initiation and maintenance of spermatogenesis is the gonadotropin follicle-stimulating hormone (FSH) secreted by the pituitary gland. Prenatally and prepubertally, FSH stimulates Sertoli cell (SC) proliferation and this determines their final number and the potential for spermatogenic output because 1 SC can only serve for a defined number of germ cells (9). Once meiosis has started, subsequent germ cell development is independent of FSH. In adulthood, FSH homeostasis is regulated and maintained by the release of inhibin B from the SCs, which provide a negative feedback to FSH secretion (10, 11) in a tight endocrine feedback system with FSH levels in the reference range (1-7 IU/L), indicating normal spermatogenesis. Thus, without obstruction in the seminal tract, significantly increased FSH (>7 IU/L) is a highly valuable diagnostic parameter of testicular insufficiency (12, 13).

However, apart from these well-known phenotypes we came across a different phenotype in NOA exhibiting an apparently different endocrine pattern when displaying spermatogenic failure in the presence of normal FSH levels. Although the occurrence of azoospermia together with normal FSH levels has previously been reported (14-17), none of these studies have analyzed this specific subgroup of azoospermic men in more detail. Following this observation, we concluded that this group of patients could benefit from further in-depth evaluation. In this study we aimed at obtaining functional, morphological, and cellular insights into this group of infertile men with NOA and FSH within the regular range, as this peculiar phenotype might require a modified treatment compared with those with FSH upregulation.

## Patients and Methods

### Patient Cohorts

**Figure 2.**
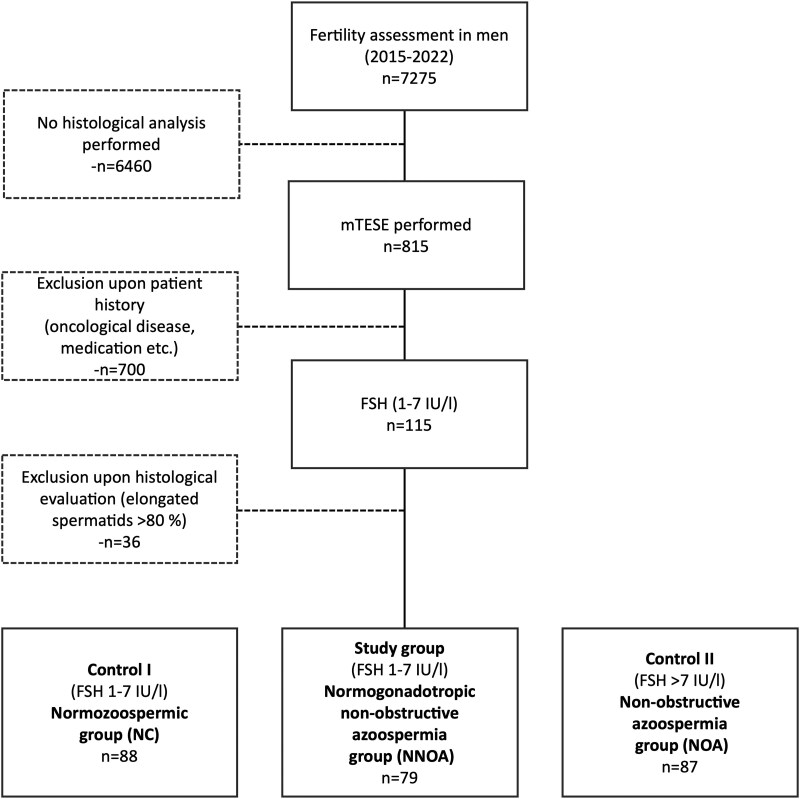
Selection of the study cohort. The flowchart depicts the selection process of infertile men with nonobstructive azoospermia (NOA) and FSH levels within regular range (1-7 IU/L) forming the study group (normogonadotropic nonobstructive azoospermia group; NNOA; n = 79). The normozoospermic group (NC; n = 88; FSH 1-7 IU/L) and hypergonadotropic nonobstructive azoospermia group (NOA; n = 87, FSH >7 IU/L) served as control groups and were accordingly selected.

We retrospectively selected infertile men from our patient database Androbase (18) who had visited the Centre of Reproductive Medicine and Andrology, Münster, Germany, for fertility workup between 2015 and 2022. The study group of interest were men with unexplained azoospermia and regular FSH values, the normogonadotropic NOA (NNOA) group. The term “unexplained” means that no strong etiologic factors are identified in somatic, ultrasound, endocrine, or genetic examinations (details see below). Two further groups served as controls: (1) hypergonadotropic NOA group, and (2) normozoospermic group (Fig. 2).

A prerequisite for the NNOA and NOA groups was that (m)TESE had been performed, and that histological samples and their respective evaluation were available. To rule out further etiologic factors contributing to the impaired fertility status and having a highly selected group of unexplained/idiopathic men, the following exclusion criteria were applied equally to all 3 groups: genetic pathology (karyotype analysis, Y-chromosome microdeletion, and gene-panel analysis including up to 26 genes known to be causative for azoospermia), oncological disease, and medication affecting spermatogenesis. To exclude men with idiopathic hypogonadotropic hypogonadism we also excluded patients with luteinizing hormone (LH) ≤1 IU/L and testosterone ≤8 nmol/L. To exclude obstructive origin, men with ejaculate volume less than 1.5 mL and patients with more than 80% elongated spermatids in the testicular histology sample were excluded. We identified (1) 79 infertile men for the study group (NNOA group) with NOA and FSH levels within normal range (1-7 IU/L), (2) 87 infertile men for the azoospermic control group (NOA group) with NOA and upregulated FSH levels (>7 IU/L), and (3) 88 normozoospermic men for the normozoospermic control (NC) group with a total sperm count ≥39 Million (Mio)/ejaculate and physiological FSH serum level (1-7 IU/L) (Fig. 2).

All patients provided written informed consent for the use and evaluation of the clinical data and biopsy samples. The study was carried out in accordance with the protocols approved by the Ethics Committee of the Medical Faculty and the state medical board (Az. 2013-255-f-S).

### Clinical Workup and Laboratory Analyses

Routine clinical workup comprised physical examination including somatic parameters, testicular ultrasound (high-frequency 12-15 MHz linear transducer), semen analysis, and hormonal measurement (FSH, LH, testosterone, estradiol, inhibin B (INHB), anti-muellerian-hormone (AMH).

Serum testosterone levels as well as FSH, LH, and estradiol were measured with a 2-step chemiluminescent microparticle immunoassay using an Abbott ARCHITECT i1000SR machine.

Mean intra-assay coefficients of variation (CVs) were below 2% and mean interassay CVs below 5%. Exact procedures were previously published (19). For inhibin B (inhibin B GEN II ELISA, A81303, Beckman Coulter, RRID:AB_2827405) and AMH (AMH GEN II ELISA, A79765, Beckman Coulter, RRID:AB_2800500) commercially available enzyme-linked immunosorbent assays were used, according to the manufacturer's instructions. Mean intra-assay CVs were 4.0% and mean interassay CVs were 4.3% for AMH, and 3.9% and 6.0% for inhibin B, respectively.

Ejaculates were obtained by masturbation after sexual abstinence for 2 to 7 days at 2 different time points. Semen analyses was carried out in accordance with the World Health Organization manual (20). Azoospermia was diagnosed when no spermatozoa were present in the ejaculate after centrifugation in both replicates.

### Testicular Tissue Preparation for Histology and Ploidy Analysis

All patients with azoospermia underwent bilateral mTESE, as described previously (21). Sperm retrieval rate (SRR) was considered positive when at least 1 spermatozoon was present (19).

Testicular biopsies for histological analyses were taken bilaterally and multilocularly during surgery. For research purposes, 1 biopsy per testis, frozen at −80 °C, and 1 additional biopsy fixed in Bouin's solution, embedded in paraffin, sectioned, and stained with hematoxylin, eosin, and periodic acid–Schiff for histological analysis were available. Histological evaluation was based on the percentage of tubules with elongated spermatids present, round spermatids, spermatocytes, spermatogonia, SCO, or complete hyalinization (“tubular shadows”). In 2 cases we used the histological evaluation of the left testis, as we had no information available for the right side.

The status of spermatogenic progress was additionally determined via flow cytometrical ploidy analysis according to a previously published protocol (22). In brief, approximately 10 to 15 mg of the frozen testicular biopsy samples (native preparations) were thawed and transferred into propidium iodide staining buffer. Biopsies were mechanically dissected, aspirated, homogenized, and centrifuged. The supernatant was incubated for 15 minutes in the dark and further analyzed using a CytoFLEX S flow cytometer. DNA content of singlets within the propidium iodide solution was detected with a 488 nm laser and a 690/50BP PC 5.5 filter. Resulting peaks during measurement gave information concerning the ploidy of the cell content and included 4C (G2 spermatogonia, primary spermatocytes), 2C (Leydig cells, SCs, somatic cells, G1 spermatogonia, secondary spermatocytes), 1C (round spermatids, elongating spermatids), and CS (spermatozoa) peaks (23). The specific proportions and their combination were used as a read-out of the spermatogenic progress.

### Statistics

All statistics were calculated using R (R, 2021) in RStudio (RStudio, 2021) with a set error probability of *P* < .05. Individual parameters were expressed as mean and SD. Normal distribution was analyzed through the Shapiro–Wilk normality test and comparisons between study and control groups were conducted using the nonparametric Mann–Whitney U test. Associations between endocrine values were investigated using the nonparametric Spearman rank correlation test. Differences in frequencies of the most advanced germ cell types were determined using the Fisher exact test.

Inhibin B levels were determined for all patients. Notably, 37 out of 87 patients in the NOA group had inhibin B values below the limit of detection, which is why 42.5% of patients could not be assigned a distinct value. A representative inhibin B value of 7.07 pg/mL was assigned for the respective patients and included in the calculation.

## Results

### Rationale for Assignment

We retrospectively selected infertile men who had visited our center for fertility workup from 2015 to 2022. We identified a subgroup of 79 men who were classified as nonobstructive azoospermic (but did not present with the expected FSH upregulation [1-7 IU/L], ie, being designated as NNOA). Two different control groups with physiological endocrine regulation were assigned: patients with NOA with elevated FSH serum levels (NOA group; control for spermatogenic failure), and patients with normogonadotropic normozoospermia (NC group; control for normal hormonal regulation) (Fig. 2).

### Somatic and Reproductive Parameters

Relevant clinical data of the 3 groups were comparatively evaluated. Medians and ranges of somatic and reproductive parameters and hormones are presented in Table 1. The median age of the NNOA group was 32 (22-57) years and significantly different to the mean age of the NOA group (35 [20-52] years; *P* = .001) and that of the NC group (35 [23-52] years; *P* = .003).

**Table 1.** Reproductive parameters of the study and control groups

	NC group n = 88	NNOA group n = 79	NOA group n = 87
**Somatic parameters**			
Age (years)	35** *^a^* **(23-52)** n = 88	32 (22-57) n = 79	35*** *^b^* **(20-52)** n = 87
Bitesticular volume (mL)	41*** *^a^* **(18-99)** n = 88	30 (12-89) n = 79	18*** *^b^* **(4-56)** n = 87
**Semen parameters**			
Ejaculate volume (mL)	3.9 (1.6-8) n = 88	3.8 (1.7-20.4) n = 78	3.8 (1.1-9.9) n = 84
Total sperm count (10^6^)	187.4*** *^a^* **(41.6-1046.5)** n = 88	0 (0-0) n = 78	0 (0-0) n = 84
α-glucosidase (>20 mU/ejaculate)	100.1*** *^a^* **(29.3-447.8)** n = 84	49.9 (5.1-940.6) n = 77	52.7 (4.7-234.4) n = 81
Fructose (>13 µmol/ejaculate)	61.4 (7.3-212.1) n = 86	59.4 (1.6-491.1) n = 78	60.9 (14.6-218.9) n = 84
Zinc (>2.4 µmol/ejaculate)	8.2 (0.5-22.4) n = 86	8.1 (0.4-58.7) n = 77	7.2 (1-31.9) n = 84
**Endocrine profile**			
FSH (1-7 IU/L)	2.7*** *^a^* **(1.1-6.7)** n = 88	5 (1.2-7) n = 79	22*** *^b^* **(10.5-63.8)** n = 87
LH (2-10 IU/L)	2.4*** *^a^* **(1-7.3)** n = 88	3.3 (1.3-7.1) n = 79	7.3*** *^b^* **(2.6-14.5)** n = 87
Testosterone (>12 nmol/L)	17.3* *^a^* **(10.1-39.5)** n = 88	15.6 (4.3-34.5) n = 79	13.2 (5.3-33.7) n = 87
Estradiol (<250 pmol/L)	81 (39-172) n = 85	90.5 (38-185) n = 72	86 (43-245) n = 83
Inhibin B (>100 pg/mL)	230.3*** *^a^* **(62.2-449)** n = 88	129.1 (16.9-365.6) n = 75	11.5*** *^b^* **(7.07-128.1)** n = 80
AMH (1.3-14.8 ng/mL)	7.4** *^a^* **(2.7-18)** n = 33	5.6 (0.8-52.1) n = 78	3.3*** *^b^* **(0.2-50.2)** n = 85

Depicted values include the normogonadotropic nonobstructive azoospermia group (NNOA), the normozoospermic group (NC) and the hypergonadotropic nonobstructive azoospermia group (NOA). Data were obtained from the in-house data base Androbase, the clinical patient database at the CeRA. The number of patients were specified due to nonavailability of samples in some cases. Reference ranges are depicted below the respective parameters. Statistics: *P ≤ .05; **P ≤ .01; ***P ≤ .001.

^a^NNOA group vs NC group.

^b^NNOA group vs NOA group (Mann–Whitney U test).

Median bitesticular volume in the NNOA group was 30 (12-89) mL, which differed significantly compared with that of the NOA group (18 [4-56] mL) and that of the NC group (41 [18-99] mL) (Table 1).

Ejaculate volumes did not differ among the groups (NNOA: 3.8 [1.7-20.4] mL; NOA: 3.8 (1.1-9.9) mL; and NC: 3.9 (1.6-8) mL, median and range, respectively). To rule out obstructive origin of azoospermia, accessory gland markers (α-glucosidase, fructose, and zinc) were evaluated. Significant differences were only detected for α-glucosidase values in patients with NNOA and NC patients (*P* < .001) (Table 1).

### Endocrine Profiles

**Figure 3.**
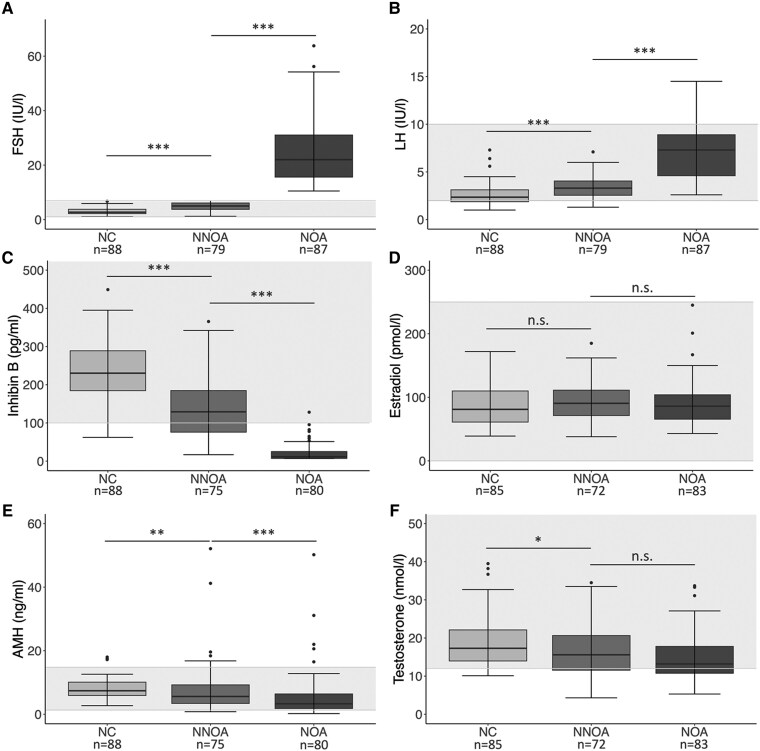
Evaluation of relevant reproductive hormones between study and control groups. Reproductive serum hormone levels of the normogonadotropic nonobstructive azoospermia (NNOA), the normozoospermic (NC), and the hypergonadotropic nonobstructive azoospermia group (NOA). FSH (A), LH (B), inhibin B (C), estradiol (D), AMH (E), and testosterone (F). Exact numbers of included patients are specified. The boxes represent the first, second (median) and third quartiles. Outliers are depicted as dots above the whiskers. The gray background represents the normal range for the respective hormone. Statistics: *P ≤ .05; **P ≤ .01; ***P ≤ .001; n.s. *P* > .05 (Mann–Whitney U test).

Median FSH was 5 (1.2-7) IU/L for patients with NNOA, 22 (10.5-63.8) IU/L for with patients NOA, and 2.7 (1.1-6.7) IU/L for NC patients. FSH values within the NNOA group were thus lower than the NOA group (*P* < .001) and higher than the NC group (*P* < .001) (Table 1 and Fig. 3A).

Median values for LH were 3.3 (1.3-7.1) IU/L for the NNOA group, 7.3 (2.6-14.5) IU/L for the NOA group, and 2.4 (1-7.3) IU/L for the NC group and significantly different respectively (*P* < .001) (Table 1 and Fig. 3B).

Median inhibin B values were 129.1 (16.9-617.2) pg/mL for the NNOA group and 230.3 (62.2-449) pg/mL for the NC group. Regarding the NOA group, all patients except for 1, had inhibin B values below the normal range (>100 pg/mL) with a median of 11.5 (7.07-128.1) pg/mL. No distinct inhibin B value could be determined for 37 patients with NOA, which is why a representative value of 7.07 pg/mL was assigned. Comparisons between all groups revealed significant differences (*P* < .001) (Table 1 and Fig. 3C).

For AMH, median values were 5.6 (0.8-52.1) ng/mL in the NNOA group, 3.3 (0.2-50.2) ng/mL in the NOA group, and 7.4 (2.7-18) ng/mL in the NC group. Although significant differences were detected between the NOA group (*P* < .001) and the NC group (*P* = .01), the majority of AMH values for all 3 groups was within normal range (1.3-14.8 ng/mL) (Table 1 and Fig. 3E).

Median testosterone was 15.6 (4.3-34.5) nmol/L in patients with NNOA, 13.2 (5.3-337) nmol/L in patients with NOA, and 17.3 (10.1-39.5) nmol/L in the NC group. Testosterone values within the NNOA group were significantly lower than the NC group (*P* = .036). No differences were detected between the NNOA and NOA groups (*P* = .081) (Table 1 and Fig. 3F).

For estradiol, median values were 90.5 (38-185) pmol/L in patients with NNOA, 86 (43-245) pmol/L in patients with NOA, and 81 (39-172) pmol/L in the NC group. There were no differences when comparing the NNOA group to the NOA group (*P* = .462) and also not when comparing the NNOA group with the NC group (*P* = .126) (Table 1 and Fig. 3D).

**Figure 4.**
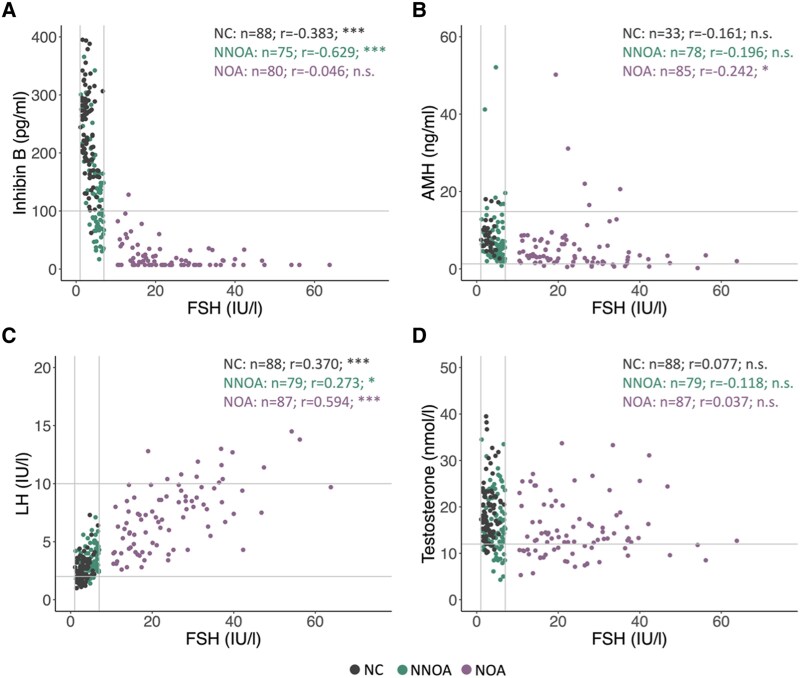
Correlations between FSH and distinct hormones. Serum levels of FSH were correlated with inhibin B (A), AMH (B), LH (C) and testosterone serum (D) levels within the normogonadotropic nonobstructive azoospermia group (NNOA; green), the normozoospermic group (NC; grey) and the hypergonadotropic nonobstructive azoospermia group (NOA; purple). Each dot represents an individual patient. The gray lines depict the normal range for the respective hormones. Number specifications of included patients are given. Statistics: *P ≤ .05; **P ≤ .01; ***P ≤ .001; n.s. *P* > .05 (nonparametric Spearman rank correlation test).

In order to identify differences in FSH signaling, correlation analysis was conducted for the respective FSH values together with LH, inhibin B and AMH. For the NNOA group, FSH correlated negatively with inhibin B (r = −0.629, *P* < .001) (Fig. 4A) and positively with LH (r = 0.273, *P* < .001) (Fig. 4C). Regarding the NOA group, negative correlation between FSH and AMH was observed (r = −0.242, *P* = .02) (Fig. 4B), while FSH and LH were positively correlated (r = 0.594, *P* < .001) (Fig. 4C). The NC group showed a negative correlation for FSH and inhibin B (−0.383, *P* < .001) (Fig. 4A) and a positive correlation for FSH and LH (r = 0.370, *P* < .001) (Fig. 4C).

### Histological Evaluation

In patients with NNOA and NOA, mTESE delivered histological samples, and SRRs were available. We assigned subgroups within the NNOA and NOA cohorts upon histological classification according to the most advanced germ cell type present (including elongated and round spermatids) in the right testis (Table 2), in order to detect possible differences in cell development.

**Table 2.** Histological stratification depending on most advanced germ cell type

	NNOA group n = 79	NOA group n = 87
Total sperm count (×10^6^)	0.0 (±0.0)	0.0 (±0.0)
**Most advanced germ cell type**		
Elongated spermatids	40 (50.6%)***	23 (26.4%)
Round spermatids	16 (20.3%)***	7 (8.0%)
Spermatocytes	15 (19.0%)***	15 (17.2%)
Spermatogonia	0 (0%)	2 (2.3%)
Sertoli cell only	8 (10.1%)**	39 (44.8%)
Tubular shadow	0 (0%)	1 (1.1%)
Positive sperm retrieval rate	47 (59.5%)***	35 (40.2%)

Results are based on evaluations of periodic acid-Schiff–stained testicular biopsies and positive sperm retrieval rate (SRR). Values are depicted as total numbers and respective percentages for the normogonadotropic nonobstructive azoospermia group (NNOA) and the hypergonadotropic nonobstructive azoospermia group (NOA). Total sperm count is given as mean ± SD. Statistics: *P ≤ .05; **P ≤ .01; ***P ≤ .001; n.s. *P* > .05 (Fisher's exact test).

Results are presented as median and range.

Concerning the NOA group, the majority of the patients were categorized as SCO (n = 39, 44.8%). Further, 23 men (26.4%) presented with elongated spermatids, 7 men (8.0%) with round spermatids, and 15 men (17.2%) with spermatocytes as their most advanced germ cell type. Spermatogonial arrest was found in 2 patients with NOA (2.3%) and tubular shadow in 1 man with NOA (1.1%). In contrast, about half of patients with NNOA showed elongated spermatids within their biopsy samples (n = 40, 50.6%). Furthermore, 16 patients (20.3%) in the NNOA group presented with round spermatids as their most advanced germ cell type, while 15 further patients (19.0%) showed meiotic arrest at the spermatocyte level. SCO was detected in the biopsies of 8 men (10.1%). No spermatogonial arrest or tubular shadow was found in the histological samples of the NNOA group. The proportion of the patients with maturation arrest thus differed between the 2 groups.

Further differences were detected concerning the SRR, with spermatozoa present in biopsy samples of 47 patients (59.5%) within the NNOA group and 35 patients (40.2%) within the NOA group (Table 2).

### Ploidy Analysis of Testicular Tissue

The ploidy of testicular cells was compared between 6 selected patients per NNOA and NOA group, respectively, within the previously described histologically defined subgroups. In order to ensure comparability and normalization, total singlet numbers from the individual cytometric measurements were transformed into percentages of all events counted for the 4C (G2 spermatogonia, primary spermatocytes), 2C (Leydig cells, SCs, somatic cells, G1 spermatogonia, secondary spermatocytes), 1C (round spermatids, elongating spermatids), and CS (spermatozoa) counts.

**Figure 5.**
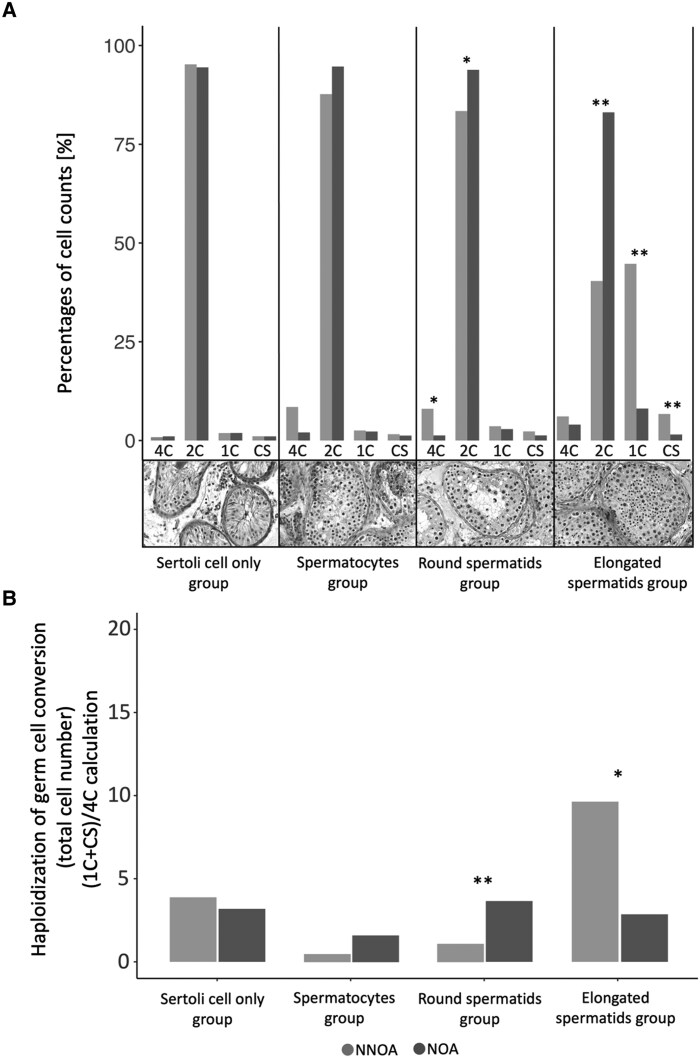
(A) Nuclear DNA content of testicular tissue within the histological subgroup detected by ploidy analysis. Subgroups were formed according to the most advanced cell type: Sertoli cell only, spermatocytes, round spermatids, and elongated spermatids. Comparisons within each subgroup (n = 6) were made between the normogonadotropic nonobstructive azoospermia group (NNOA; light grey) and the hypergonadotropic nonobstructive azoospermia group (NOA; dark grey), adding up to a total of 48 ploidy analyses conducted via flow cytometry. The different peaks represent different cell populations as a percentage share within the examined sample (4C = G2 spermatogonia, primary spermatocytes; 2C = Leydig cells, Sertoli cells, somatic cells, G1 spermatogonia, secondary spermatocytes; 1C = round spermatids, elongating spermatids; CS = condensed spermatids). Histologic images each show a patient from the subgroup based on their categorization depending on the most advanced germ cell type. Data are displayed as medians (top of the bar). Statistics: *P ≤ .05; **P ≤ .01; ***P ≤ .001 (Mann–Whitney U test). (B) Haploidization of germ cell conversion. Flow cytometry was conducted to analyze the nuclear DNA content of testicular tissue within the formed subgroups based on prior histological evaluation of testicular biopsies. Haploidization of germ cell conversion was calculated by summing up total numbers of singlets of the haploid cell types (ie, spermatozoa, and spermatids [1C + CS]), which then were divided by diploid G2 spermatogonia and primary spermatocytes (4C) (ie, (1C + CS)/4C), thus giving information regarding meiotic efficiency. Data are displayed as medians (top of the bar). Statistics: *P ≤ .05; **P ≤ .01 (Mann–Whitney U test).

Concerning the round spermatids subgroup, significantly more double-diploid singlets (4C%) were present within the biopsies of the NNOA group compared with the NOA group (*P* = .03). However, more diploid singlets (2C%) were counted for the NOA group (*P* = .04). Regarding the elongated spermatids subgroup, more haploid singlets were detected in the tissue of NNOA patients (1C%: *P* = .01; CS%: *P* = .02). Again, more diploid singlets (2C%) were counted for the NOA group (*P* = .01) than for the NNOA group (Fig. 5A).

The efficiency of germ cell conversion was defined as the number of elongated spermatids and spermatozoa (1C + CS), divided by the number of G2 spermatogonia and primary spermatocytes (4C) (ie, (1C + CS)/4C). Germ cell conversion efficiency was significantly different between the NNOA and NOA group, with regard to the round spermatids (*P* = .01) and elongated spermatids (*P* = .04) subgroups (Fig. 5B).

## Discussion

### Identification of a Specific NNOA Subgroup

In NOA, no spermatozoa can be detected in the ejaculate, pointing to a severe disturbance in gamete production as such (24). Disturbances of the spermatogenic differentiation process that result in NOA are known to provoke elevated FSH levels. Increased FSH levels, therefore, mirror truncated spermatogenesis, pointing to a disruption of the feedback loop along the hypothalamo–pituitary–gonadal (HPG) axis (6) and therewith present a highly valuable diagnostic parameter.

There is some previous evidence of a different endocrine phenotype, namely men with nonobstructive azoospermia and normal FSH serum level (14-17, 25-30). We also observed a substantial number of patients with NOA exhibiting a different endocrine phenotype. Specifically, these patients showed normogonadotropic FSH (1-7 IU/L) levels, but otherwise the same pattern of disrupted spermatogenesis, resulting in normogonadotropic NNOA. In this study, we aimed to characterize this peculiar group of patients in depth and compare them with patients with hypergonadotropic NOA and normozoospermia.

### Differences in Somatic and Reproductive Parameters

Significant age differences were detected between the study (NNOA) and both control groups (NOA and NC). However, within this study, this difference was assessed as biologically irrelevant, as the men of all groups were in the same reproductive life phase, and the age difference, thus, cannot be reasoned to be causative for the phenotype observed. Bitesticular volume was lower in the NNOA study group than in the NC control group. This is consistent with the literature, since a testicular disturbance resulting in azoospermia is often accompanied by reduced testicular volume resulting from reduced germ cell maturation (31, 32). Interestingly, patients of the NOA group also showed lower bitesticular volumes than patients of the NNOA group, suggesting the latter to be in an intermediate, less affected state.

Furthermore, patients of the NNOA group were similar to patients with NOA with regard to other aspects of NOA, be it ejaculate volume or accessory glands markers that can link towards obstruction (33, 34). Altogether, both patients with NNOA and patients with NOA met the requirements for ruling out an obstruction of the seminal ducts, especially since ultrasound results were also included in the diagnoses and since patients with more than 80% elongated spermatids in the testicular histology sample were excluded.

### Differences in Endocrine Regulations

FSH is a particularly important hormone that is necessary for the maintenance of the HPG axis and functional spermatogenesis (6, 7, 35). Mathematically, values for FSH of the NNOA group differed from the NC group. However, physiologically, both have to be classified as similar from a clinical point of view, as all FSH values were within normal range (1-7 IU/L). Based on the study design, FSH values of the NNOA and NOA groups differed substantially; however, FSH levels are continuous and there is no distinct absolute threshold that separates normal and elevated FSH values (36). Although we used >7 IU/L as a cutoff definition for elevated FSH, it is important to note that in our control group (NOA), the lowest measured FSH level was 10 IU/L, with most patients having a level considerably higher than 7 IU/L.

In order to examine whether the dysfunction leading to the NNOA phenotype is caused at the gonadotropin level, LH was examined next to FSH. Based on this, a first indicator for the functionality of the hypothalamus and the pituitary gland could be inferred, as the pituitary gland is the site of gonadotropin production (6). We are aware that this gives a limited hint of the pituitary function, as dynamic testing such as the gonadotropin-releasing hormone test could even reveal more comprehensive data; however, these data were not available as this is only performed on demand in our center. Although the groups differed in their LH values, the levels of all 3 groups were predominately within normal range. Thus, no dysfunction at the LH level was detected, which could have been a causative factor for the development of the NNOA phenotype. To rule out the possibility that the lack of FSH upregulation in patients with NNOA is related to sex steroids, testosterone and estradiol levels were evaluated. As no relevant differences were detected in this respect, a causality can be precluded.

Inhibin B and AMH are both secreted by SCs and represent important signals in testicular development and function. Although AMH is a product of immature SCs produced prepubertally (37, 38), inhibin B is a key regulatory factor in the adult testis and has an important function in the feedback loop of the HPG axis (6, 39). In adulthood, missing to low AMH expression shows a normal functional differentiation of the SCs, as does the production of inhibin B. Based on the AMH values, a maturation defect of SCs can be excluded. Although there were significant differences in the comparison of AMH values between all three groups (NNOA, NOA, and NC), the majority of all patients within these groups displayed values within the normal range. Inhibin B values were previously found to become progressively lower in men with increasing spermatogenic dysfunction and even sometimes undetectable in cases of azoospermia (40). This falls in line with observations made within this study, since no clear value for inhibin B could be measured in 42.5% of the patients with NOA, as values were below the detection limit, while the remaining patients with NOA exhibited inhibin B values below the normal range. Of particular interest, however, were the findings in the NNOA group. Here, the majority of inhibin B values were within normal range, regardless of the fact that all patients in this group were azoospermic. An inverse association between FSH and inhibin B in fertile and infertile men was also previously described (41-43).

Taken together, the comparative endocrine analyses revealed normogonadotropic FSH levels remaining within normal range in the NNOA group, although—compared with the NOA and NC group—the FSH values were intermediate as were LH, inhibin B, AMH and testosterone values for the patients with NNOA. However, in order to obtain an even better impression of endocrine interactions within the NNOA group, correlation analyses were additionally performed to check whether there were any differences in hormonal responses when also examining the NOA and NC groups. Based on these correlation analyses, it was observed that the NNOA and NC groups appeared to behave in a highly similar way on an endocrine basis. Both groups showed correlations of FSH and inhibin B levels and of FSH and LH, while no correlations were detected between FSH and AMH and testosterone. The NOA group, on the other hand, behaved differently according to these analyses, while the correlation values even appeared to mostly overlap for patients with NNOA and patients with NC. From this observation, it can be assumed that the feedback loop of the HPG axis regarding the NNOA group must presumably be functional, since the response is comparable to that of healthy normozoospermic men.

In principle, an endocrine disorder in patients with NNOA can likely be ruled out, as FSH, LH, and testosterone appear normally regulated. (Men with idiopathic hypogonadotropic hypogonadism [IHH] were excluded, based on the exclusion criteria mentioned above.) In the NNOA group, inhibin B levels below standard were detected in 31 out of 75 measured blood samples, making up approximately 41%. The remaining patients, however, exhibited normal inhibin B levels, despite of being azoospermic. This aberrant inhibin B response in NNOA patients, compared with patients with NOA and NC, suggests that SCs, which are responsible for inhibin B production, may have a direct impact on the pathogenesis of the NNOA phenotype, which is why they should be allocated a more extensive focus.

### Histological Evaluations of Testicular Tissue in Patients With NNOA and NOA

Histological findings from the NNOA group exhibited on average more differentiated germ cell stages and higher positive SRRs than the NOA group. Based on this observation, it appears that azoospermic patients with normal FSH levels are more likely to present elongated spermatids in their biopsy samples. Quantitatively occurring—however still incomplete—spermatogenesis was therefore more frequently observed within the NNOA group than in the NOA group, in which the majority of patients presented as SCO. Nevertheless, normal FSH levels cannot automatically be considered as a predictor for the presence of spermatozoa in the biopsy samples, as the SRR was still negative in about 40% of patients with NNOA. This observation is also in line with previous findings stating that FSH, as well as inhibin B and testicular volume, cannot predict the presence of spermatozoa with certainty (44-48). In principle, however, all these parameters can be used to obtain a potential probability of a positive SRR. Since patients with NNOA generally exhibited more germ cells in their testicular biopsies than patients with NOA, this presumably also explains the higher average testicular volume detected in the NNOA group. Nevertheless, all these predispositions and processes that seem to operate more effectively in the NNOA group still could not provide spermatozoa in the ejaculate. This is probably because the few developed elongated spermatids or the resulting spermatozoa are not intact and thus degraded by phagocytotic and/or apoptotic processes in SCs, the epididymis, or the vas deferens (49-51).

FSH is inversely associated with the quantity of spermatogonia (ie, a grossly reduced number or the absence of spermatogonia leads to increased FSH values). In line with this, normal numbers of spermatogonia in the testes are reflected by normal FSH levels (52, 53). Furthermore, FSH levels are also related to the proportion of seminiferous tubules of the SCO phenotype (16). Since no spermatogonia could be found in the testis samples of a large number of patients within the NOA group, this could contribute to the observed elevated FSH values. In contrast, spermatogonia were found in the majority of patients in the NNOA group, thus this could be a factor in preventing FSH upregulation.

### Ploidy Analysis Revealed Postmeiotic Differences Between NNOA and NOA Patients

Our interest was in detecting the spermatogenic dynamics (ie, at which state those might be disrupted). As ploidy analysis uses large fragments of the testicular tissues, it has the advantage to integrate these processes. Additionally, it provides the most important information on spermatogenic progress, as via the 4C peak (transition/meiosis, double diploid cells) and the 1C peak (haploidization, haploid cells), the relevant processes can be easily determined and related to each other, pointing to potential disruption of germ cell maturation. Ploidy analysis identifies the DNA content of testicular cells and enables to analyze a substantial amount of tissue integrating large cell numbers (22, 23, 54).

Generally, histological findings were consistent with the ploidy results and thus corroborated the correct classification of the patients with NNOA and NOA into subgroups along the histological evaluation. Rarely, samples were found to contain even further advanced germ cell types after ploidy analysis than detected by histology only, likely because the samples integrate a much larger proportion of the testicular tissue than a histological section can provide (7).

Histologically, we discriminated between hypospermatogenesis, spermatogenic arrest (classified upon the level at which germ cell differentiation is stopped), SCO, and tubular atrophy. We found that men in the NNOA and NOA group respectively differed regarding the proportion of maturation arrest. By interpreting the results, it is of particular importance to be aware of the different arrest stages, and their endocrine regulation (Fig. 1). Interestingly, when analyzing the cellular composition of the testicular tissues, no differences were found between the NNOA and the NOA group before meiosis (ie, in the spermatocyte and SCO subgroups). Only after haploidization in the round spermatid and elongated spermatid subgroups were differences detected. This means that disparities in spermatogenic progress became only apparent in patient subgroups in which meiosis was complete (ie, the meiotic processes were obviously unaffected by the observed endocrine phenotype). This is consistent with the reported function of FSH for the premeiotic/meiotic development of germ cells. When FSH levels are normal, these parts of differentiation should also be passed normal (6, 35). Therefore, the analyzed samples are not in a state of meiotic arrest, as this step has been passed successfully.

In line with this, the germ cell conversion efficiency was also only different between the NNOA and NOA round spermatid and elongated spermatid subgroups (ie, the subgroups in which haploidization took place). Haploidization depends on the meiotic production of 4 round spermatids from 1 spermatocyte and thus 1 spermatogonia (6, 54). In particular, the elongated spermatids subgroup revealed that haploidization and meiotic efficiency appears less affected and more effective in patients with NNOA than in patients NOA.

The novelty of our findings lies in the observation that many patients with NNOA who presented with FSH levels within regular range, seem—although considered azoospermic—to have—at least from a histological point of view—the ability of completing spermatogenic progress up to the stage of elongated spermatids. This points to a potential mechanism that has not yet been fully explored/understood in patients with azoospermia diagnosis and normal FSH. The frequency of the NNOA phenotype is not rare at all, in contrast to meiotic arrest and normal FSH. We therefore believe that this distinct subgroup within the NNOA phenotype would benefit from further investigation, including the evaluation of genetic differences that might underlie the peculiar NNOA phenotype.

## Conclusion

We thoroughly characterized a peculiar male infertility phenotype of nonobstructive azoospermic men presenting FSH levels within normal range.

This is probably not due to a malfunction on the hypothalamic and/or pituitary level; it appears rather likely that the failure to upregulate FSH in patients with NNOA might be due to a malfunction at the testicular cellular level. A testicular endocrine malfunction appears unlikely, as both FSH and testosterone were in the normal range regarding the NNOA cohort and the most prominent spermatogenic differences between the patients with NOA and the patients with NNOA only became apparent postmeiotically, not in the meiotic or premeiotic stage. A possible explanation is that SCs might be functionally insufficient in terms of workload management or other physiological functions. A possible hint towards this direction could be the inhibin B response in the NNOA group, as it differs from the NOA and NC group. Therefore, SC physiology and function should receive more attention in future analyses.

The observations and findings obtained point to 2 possible explanations: first, the NNOA group represents a peculiar phenotype in which a mechanism provokes spermatogenic failure different from classic NOA only at the postmeiotic level, thus leaving FSH regulation unaffected. Second, the same mechanism underlies both NNOA and NOA, but the groups consist of patients at different stages of progression of the same disorder. This second possible explanation would suit the observed differences in SRRs, which were generally higher for the NNOA group. This could be due to a progressive effect that deteriorates over time, in which case it would be necessary to refer the affected patients to mTESE as early as possible to increase the probability of finding testicular sperm.

## Outlook

Future analyses of this specific NNOA phenotype should include the screening for potential genetic differences by exome analyses and the examination of Single Nucleotide Polymorphisms (SNPs) in this cohort. In-depth investigation of SC functions and respective potential deviations should also provide more certainty regarding the development of NNOA. Furthermore, increased group size and prospective, longitudinal evaluation and the evaluation of the dynamic character of the impairment, as well as gonadotropin-releasing hormone testing could contribute to a better detection and characterization of this specific NNOA patient cohort.

## Data Availability

Some or all datasets generated during and/or analyzed during the current study are not publicly available but are available from the corresponding author on reasonable request.

## Abbreviations

FSH: follicle-stimulating hormone
HPG: hypothalamo–pituitary–gonadal
LH: luteinizing hormone
mTESE: microsurgical testicular sperm extraction
NC: normozoospermic control
NOA: nonobstructive azoospermia
NNOA: normogonadotropic nonobstructive azoospermia
SC: Sertoli cell
SCO: Sertoli cell only
SRR: sperm retrieval rate
